# Evaluation of a High Resolution Genotyping Method for *Chlamydia trachomatis* Using Routine Clinical Samples

**DOI:** 10.1371/journal.pone.0016971

**Published:** 2011-02-11

**Authors:** Yibing Wang, Rachel J. Skilton, Lesley T. Cutcliffe, Emma Andrews, Ian N. Clarke, Pete Marsh

**Affiliations:** 1 Molecular Microbiology and Infection, School of Medicine, University of Southampton, Southampton, United Kingdom; 2 Regional Microbiology Network Southampton Laboratory, Health Protection Agency, Southampton General Hospital, Southampton, United Kingdom; Charité-University Medicine Berlin, Germany

## Abstract

**Background:**

Genital chlamydia infection is the most commonly diagnosed sexually transmitted infection in the UK. *C. trachomatis* genital infections are usually caused by strains which fall into two pathovars: lymphogranuloma venereum (LGV) and the genitourinary genotypes D–K. Although these genotypes can be discriminated by outer membrane protein gene (*omp*A) sequencing or multi-locus sequence typing (MLST), neither protocol affords the high-resolution genotyping required for local epidemiology and accurate contact-tracing.

**Principal Findings:**

We evaluated variable number tandem repeat (VNTR) and *omp*A sequencing (now called multi-locus VNTR analysis and *omp*A or “MLVA-*omp*A”) to study local epidemiology in Southampton over a period of six months. One hundred and fifty seven endocervical swabs that tested positive for *C. trachomatis* from both the Southampton genitourinary medicine (GUM) clinic and local GP surgeries were tested by COBAS Taqman 48 (Roche) PCR for the presence of *C. trachomatis*. Samples tested as positive by the commercial NAATs test were genotyped, where possible, by a MLVA-*omp*A sequencing technique. Attempts were made to isolate *C. trachomatis* from all 157 samples in cell culture, and 68 (43%) were successfully recovered by repeatable passage in culture. Of the 157 samples, 93 (*i.e.* 59%) were fully genotyped by MLVA-*omp*A. Only one mixed infection (E & D) in a single sample was confirmed. There were two distinct D genotypes for the *omp*A gene. Most frequent *omp*A genotypes were D, E and F, comprising 20%, 41% and 16% of the type-able samples respectively. Within all genotypes we detected numerous MLVA sub-types.

**Conclusions:**

Amongst the common genotypes, there are a significant number of defined MLVA sub-types, which may reflect particular background demographics including age group, geography, high-risk sexual behavior, and sexual networks.

## Introduction

Genital chlamydia infection is the most frequently diagnosed sexually transmitted infection in the UK. The number of infected individuals with chlamydia identified through testing continues to increase annually (http://www.hpa.org.uk/Topics/InfectiousDiseases/InfectionsAZ/STIs/STIsAnnualData/AnnualSTISlideset/). *C. trachomatis* is traditionally divided into at least 15 ‘serovars’ which are linked to different disease pathologies. As the majority of women, and a significant proportion of men, diagnosed with this infection are asymptomatic, there is a large pool of prevalent undiagnosed infection which provides a reservoir for further spread (http://www.bashh.org/guidelines). The spread of infection is determined by patterns of sex partner selection, the relative risk of infection is associated with particular social networks and the existence of bridge populations [1]. Genital chlamydial infections are caused by two ‘pathovars’: lymphogranuloma venereum (LGV: L1,L2 and L3 - invasive and disseminating) and infections associated with serovars D to K, which are non-invasive and can cause epididymitis, cervicitis, urethritis, salpingitis and pelvic inflammatory disease [2]. Sequelae following infection can be severe, such as infertility in women (including pelvic inflammatory disease and ectopic pregnancy) (http://www.bashh.org/guidelines).

Standard multi-locus sequence typing (MLST) approaches which target a limited number of relatively stable and non-selected *loci* such as house-keeping genes [3], [4] have been developed for global surveillance and show a medium-level resolution across all chlamydia strains at the genotype/serovar level. But MLST as performed by Pannekoek et al [3] is insufficiently discriminating to resolve closely related *C. trachomatis* isolates. An example of the limited resolution of MLST is the grouping of LGV strains (belonging to serovars L1, L2 and L3) to a single cluster of identical MLST-type using the scheme of Pannekoek et al [3], which was developed using a small number of European isolates and the two complete published genomes available at the time [5], [6]. The more recent MLST scheme developed by Dean et al [4] is only able to discriminate L2b (the proctitis-causing variant of L2) from the rest of the LGV group, and does not discriminate between L1, L2 and L3. However, the purpose of MLST schemes in highly homogeneous bacterial populations is to describe population structure and diversity, and not to operate at the level of an ultimate strain typing scheme [7]. There is growing evidence of a small but meaningful variation at the genome level [8], therefore methods of high resolution genotyping are required to reveal more discriminating data. Such data is important for the monitoring of therapy and contact tracing including route-cause analysis, and study of sexual networks.

An “MLST” system has been described [9] that shows some promise for discriminating strains, however this does not follow the rules of MLST (exclusive use of housekeeping genes and small amplicons), therefore this should really be considered as multi-sequence typing (MST). There has been only one attempt at developing rapid, high resolution genotyping [10], which targets short, mutable sequences and thus facilitates detailed resolution of local diversity at high efficiency in short periods of time (the latter incorporating variable number tandem repeat (VNTR) analysis). VNTRs are repetitive sequences of the same nucleotide or motif, and variation in DNA polymerization errors in different strains gives rise to variation in length between strains [10]. Multi-locus VNTR analysis (MLVA) of *C. trachomatis* VNTR sites and *omp*A appeared to facilitate a highly discriminatory genotyping survey of *C. trachomatis* from clinical samples from Aarhus in Denmark [10]. To evaluate this scheme unbiased, the MLVA-*omp*A approach previously (and incorrectly) called “*omp*1-VNTR” [10] requires independent analysis on clinical samples in other laboratories. The original Aarhus study [10] which utilizes four PCR targets returned data for all samples collected, whereas most studies are only successful in analyzing a percentage of samples collected, and the scheme has not been assessed independently using routine clinical samples to study epidemiology in a typical host population. This scheme has been compared to the MLST scheme of Pannekoek et al [3] and the MST scheme of Klint et al [9] in a retrospective study of a small number highly selected set of samples which had been cell-culture adapted in Moscow collected in 2005 [11]. Amplification conditions were highly optimized as the chlamydial DNA was prepared from semi-purified elementary bodies: the approach in the Moscow study is therefore not applicable to routine clinical samples.

Our aim was to evaluate independently the MLVA-*omp*A approach. To facilitate this we used routine clinical swab samples from women in the local *C. trachomatis* diagnostic laboratory to test the success rate of MLVA-*omp*A analysis for all four markers. Our selection of samples positive for *C. trachomatis* was a commercial nucleic acids amplification technology (NAAT) test (COBAS Taqman 48, Roche) which uses highly conserved DNA targets. The fact that only swabs were collected resulted in men being excluded from this survey, because in the case of the COBAS Taqman 48 (Roche) system urine samples can also be processed and this is the preferred sample chosen by men. Therefore the study in Southampton was limited to women. Our approach resulted in a six month survey in which primary swab samples from women paired with DNA extracts with commercial NAATs *C. trachomatis* positive results were collected, cultured and genotyped. A far greater diversity in MLVA-*omp*A genotypes in comparison to using *omp*A alone was found in our sample set including a new genotype (which was a J variant) that could not be isolated in cell culture.

## Materials and Methods

### Clinical samples

Endocervical swabs collected between February and July 2009 from patients presenting at the GUM clinic in Southampton, and also samples referred from GP surgeries were analyzed for the presence of *C. trachomatis* using the commercial NAAT incorporating the Tecan DNA extraction system and COBAS Taqman 48 (Roche) real-time PCR protocol in the Health Protection Agency (HPA) Molecular Diagnostics Laboratory, Southampton. Crude DNA extraction, real-time PCR and analysis were performed according to the manufacturer's instructions. DNA extracts, which gave “strong” positive results according the cycle threshold (CT< or  = 30), were processed for high resolution genotyping as described below. The primary swab sample from which the DNA extract was made was retained, so that the transport medium (M4RT) could be used to inoculate tissue culture for isolation of live *C. trachomatis*. Although this real-time PCR protocol is not a validated quantitative assay, the CT values of positive extracts were taken to indicate a reasonable level of *C. trachomatis* carriage in that sample for use in further PCR and sequence analysis as well as having a reasonable likelihood of providing a culturable inoculum. As an additional criterion to qualify for the latter process, we excluded swab samples and their paired DNA extracts that were >3 days old from time of sampling, as samples older than this did not usually contain culturable inocula. DNA extracts were stored at −80°C prior to MLVA-*omp*A analysis. The paired transport medium samples were stored at 4°C for <4 days following collection prior to isolation in cell-culture. Approval by ethical committee and patient consent were not obtained as this was considered a standard evaluation of an existing method. Furthermore the samples that we used had been discarded following routine diagnostic analysis, and they were unlinked and anonymised so as to permanently protect patient confidentiality.

### Culture of isolates

The transport medium (M4RT) contained gelatin, gentamicin, and amphotericin B, and although specifically designed for transport and long term storage of viral specimens, we investigated the potential to culture *C. trachomatis* isolates directly from the clinical swabs rather than collect separate swabs for the specific purpose of this study, so as to maintain a collection of local isolates and facilitate further in-depth whole-genome and biological studies. This was possible because M4RT is designed to preserve organism integrity rather than act as a precursor to lysis, which occurs with some other commercial systems. It should be clarified that we defined the ability of an isolate from a sample to be cultured as being capable of at least two growth-passages through tissue culture, visible at each stage as inclusions by phase contrast microscopy, thus enabling a perpetuated collection of local isolates which may be used in future studies.

A 24–well tray of McCoy cells was prepared the day before infection. For infection, the cell culture medium was removed from the cells and 1 ml of the transport medium containing potential *C. trachomatis* isolates was added to a well. The 24–well tray was then centrifuged at 754×g for 30 min to facilitate infection. The transport medium was then replaced with 1 mL fresh Dulbecco's Modified Eagle Medium containing cycloheximide (1 µg/mL), gentamicin (20 µg/mL), and vancomycin (10 µg/mL), and the cells incubated at 37°C for 72 h. After incubation, if any viable *C. trachomatis* isolates were present they could be viewed by light microscopy as inclusion bodies. Positive samples were then scraped up using a sterile pipette tip, mixed with glass beads for 1 min to break open the host cells, centrifuged at 110×g for 5 min to remove any cell debris, and then the supernatant was added to an equal volume of storage buffer (4SP) and stored at −80°C.

### Amplification of VNTR and *omp*A sequences

VNTR *loci* and *omp*A sequences were amplified by PCR from the DNA extracts from positive swab samples (as determined by the COBAS Taqman 48 method) as follows: The *omp*A gene was amplified using primers PCTM3 and NRI according to Lan et al [12] (Table 1). As a contingency, alternative primers OMPSeqF and OMPSeqR were used which amplified a 718 bp fragment of *ompA* in cases where PCR of the 1,019 bp amplicon proved not possible (Table 1). Sequences encompassing the three VNTR *loci* were amplified using primers CT1291F and R, CT1299F and R and CT1335F and R according to Pedersen et al [10] (Table 1). The PCR reaction components were: 1×HF buffer, 200 µM each of dioxynucleotriphosphates G, A, T and C, 250 nM each of forward and reverse primers (Table 1), 2–4 µl of DNA sample, 0.5 µl of Phusion High-Fidelity DNA polymerase (New England Biolabs UK, Hitchin, UK), and H_2_O to final volume of 50 µl.

**Table 1:** Primer sequences for PCR of *omp*A and VNTR *loci*.

Primer name	Primer sequence (5′ – 3′)	Amplicon size^a^ (bp)	Nucleotide position^a^	Reference
PCTM3	TCCTTGCAAGCTCTGCCTGTGGGGAATCCT		779,977–780,006	Lan et al [12]
NRI^d^	CCGCAAGATTTTCTAGATTTC	1,019	778,988–779,008	Lan et al [12]
CT1291F	GCCAAGAAAAACATGCTGGT		195,536–195,555	Pedersen et al [10]
CT1291R	AGGATATTTCCCTCAGTTATTCG	225^b^	195,760–195,738	Pedersen et al [10]
CT1299F	TTGTGTAAAGAGGGTCTATCTCCA		291,758–291,781	Pedersen et al [10]
CT1299R	AAGTCCACGTTGTCATTGTACG	188	29,1945–291,924	Pedersen et al [10]
CT1335F	TCATAAAAGTTAAATGAAGAGGGACT		737,225–737,250	Pedersen et al [10]
CT1335R	TAATCTTGGCTGGGGATTCA	153	737,377–737,358	Pedersen et al [10]
OMPSeqF	GGTGTGACGCTATCAGCATGC		779,880–779,900	This study
OMPSeqR	GACCATTTAACTCCAATGTA	718	779,183–779,202	This study
CT1291inF^d^	TACAAAAGTGTTGTGATAATTC	-	195,559–195,580	This study
CT1299inR^d^	ACGAATCCTCTAAGTACGG	-	291,908–291,926	This study
CT1335inR^d^	GGATTCAACGATGATTAAGG	-	737,345–737,364	This study
OMPSeq2R^d^	TATTGGAAAGAAGCICCTAA c	-	779,417–779,436	This study

a according to D/UW-3/CX, accession number NC 000117.

b an amplicon of 510 bp was seen in some samples.

c I = inosine.

d Primers used for sequencing.

The PCR was performed using the Veriti 96 well Thermal Cycler (Applied Biosystems International, Warrington, UK). The general PCR program was: 2 min at 98°C, followed by 35–40 cycles of 20s at 98°C, 30 s at 56–64°C and 45 s at 72°C, with a final extension step of 7 min at 72°C, and then held at 16°C. For *ompA* PCR, 35 cycles and 56–58°C annealing temperature were used. The PCR completed forty cycles for the VNTRs; the annealing temperatures were 56–59°C for CT1291 and CT1335, and 62–64°C for CT1299. The PCR products were purified using Wizard SV Gel and PCR Clean-up System (Promega, Southampton, UK).

### Sequencing

Sequencing reactions on purified PCR products were carried out by a commercial company: Gene Service (http://www.geneservice.co.uk). The sequencing primers for CT1291, CT1299 and CT1335 are internal primers CT1291inF, CT1299inR and CT1335inR, respectively. The sequencing primers for *ompA* are NR1 (longer PCR) or OMPSeq2R (shorter PCR) (Table 1).

### MLVA-*omp*A sequence analysis

*Omp*A sequences were genotyped via comparison to the NCBI database via BLAST, and sequence types were assigned using reference strains according to the following strain names and accession numbers: D, D/UW-3/CX NC 000117; E, E/BOUR DQ06428; F, F/IC-CAL3 DQ064287; G, G/392 DQ064288; I, Ia/870 DQ064291; J, J/UW-36 DQ064292; K, K/UW-31 DQ064293.

VNTR sequences were analyzed manually using the original fluorescent traces and by comparison of the alphabetical sequences to those of D/UW-3/CX, accession number NC 000117. The assignment of MLVA-type was carried out according to the rules described in Pedersen et al [10], namely, a number was assigned to each different VNTR *locus* based on the differing number of mononucleotides. Therefore the three sequenced VNTR *loci* gave rise to a corresponding three-digit code according to the order: CT1335; CT1299 and CT1291. Where new MLVA types were discovered that were not defined in Pedersen et al [10], we assigned new numbers which were added to the *C. trachomatis* MLVA database (http://www.klinmik.dk/genotyping/MLVA_types.asp).

## Results

### Preliminary Pilot study

In December 2008 we began our preliminary primary study, collecting DNA samples. In the first instance we collected nine DNA samples during that month. Of these, only seven gave a full set of amplicons compatible with the VNTR and *omp*A PCR products capable of yielding sequence data. Therefore we concluded that a proportion of our samples in the main study may not yield full genotyping data. To address this possible loss of data, we designed a study in which we collected crude DNA samples paired with the primary swab used to collect the sample, this swab was preserved in virus transport medium (Micro Test M4RT Transport medium, Remel, Lenexa, Kansas, USA). From the latter, we attempted to culture live isolates of the *C. trachomatis* present in the positive swab samples. We found that swab samples lost viability if more than three days (at 4°C) had elapsed between time of sample taking and receipt following confirmation of positivity. This contingency would allow us to extract DNA from cultured isolates and enable genotyping analysis data to be added to the data collected from the crude DNA isolates.

### Clinical samples

In the six month period between February and July 2009 a total of 5339 female swab specimens were submitted to the HPA laboratory and from these 268 (4.9%) provided a positive result. From these 268 there were 111 positive samples that did not meet our inclusion criteria leaving 157 paired positive DNA extracts with swab samples which did comply with our sampling criteria. From these 157, 46 samples originated from GP surgeries and the other (111) samples originated from the GUM clinic. The age distribution of the samples demonstrated most specimens occurred in patients who were 16–24 years of age (117/157, with a median of 20 years of age in the 16–24 range; Table 2).

**Table 2:** Distribution of samples tested (n = 157) according to age and GUM clinic or GP referral.

Age range (years)	GUM	GP referral	GUM as % of total	Total
16–24	92	25	79	117
25+	19	21	48	40
Total	111	46	71	157

### MLVA-*omp*A sequence analysis

**Figure 1:**
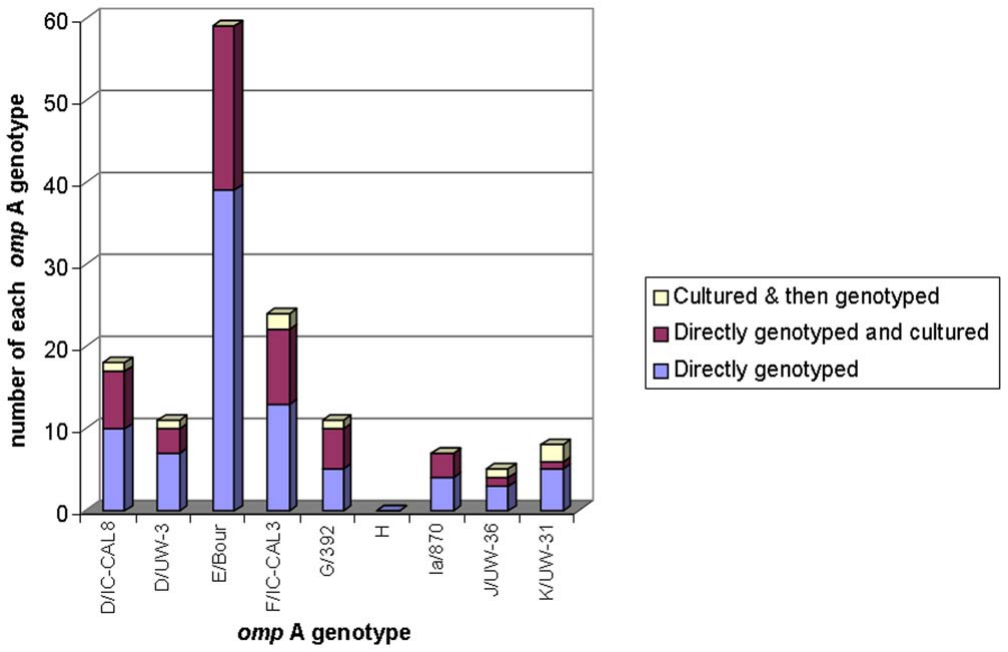
Distribution of *omp*A types discriminated according to whether they were genotyped directly from clinical samples, whether any of these were subsequently cultured, and those that could only be genotyped following culture (n = 93).

Prior to this study we tested a collection of Taq DNA polymerase types, and due to its high fidelity concluded that we should use Phusion Taq DNA polymerase (New England Biolabs UK, Hitchin, UK) for all PCR reactions. This is a high fidelity enzyme which we employed for several reasons, including to eliminate the possibility of PCR error in amplification of tandem repeats of monothymidines of greater than eleven base-pairs [13]. Direct genotyping from the DNA extracts used for the NAAT test that deployed the three VNTR *loci* and *omp*A was possible for 85 of the samples. The *omp*A sequences were aligned with 18 known reference *omp*A sequences from the GenBank sequence database [14]. The genotypes which most regularly produced a positive genotype were; D, E and F, comprising 20.4%, 40.9% and 16.1% of the genotyped positives respectively (Figure 1). Genotype D could be sub-divided into two divergent types by comparing the differing sequences available on the NCBI database. Therefore the proportion that were genotype D (20.4%) comprised 11.8% of the total which aligned with D/UW-3/CX, and 8.6% of the total which aligned with D/IC-CAL3. No genotype H was found.

Isolates were cultured (*i.e.* were capable of at least two passages through tissue culture) from 68 of the paired M4RT samples that originated as primary swab samples; 49 of these were amongst the 85 that were directly genotyped (see Table S1 for full results). All of the 89 samples that were classed as non-culturable did not show any growth in tissue on the primary passage. Of the 68 samples which were subsequently found to be culturable, use of the MLVA*-ompA* typing scheme on the primary DNA samples allowed us to identify 26 distinct types including six of genotype D; nine of genotype E; four of genotype F; four of genotype G; three of genotype Ia; and one each of J and K. Of the 19 samples that were not directly genotyped but were cultured, it was possible to extract DNA and fully genotype 8 samples (the incomplete data for all 19 isolates is shown in Table S2). To achieve this, we had to use the “short fragment” PCR and sequencing option because no result was obtainable using the long fragment option. Only where we could attain good quality sequence data for variable domains II and IV of *omp*A did we accept the identification data as accurate. Therefore we gained a further eight of nineteen cultured samples to add to our total of MLVA-*omp*A genotyped collection. Consequently we returned a total of 93 fully MLVA-*omp*A genotyped samples. The age-distribution of the 93 specimens analyzed by MLVA-*omp*A was similar to that of the 157 total specimens received, in that most specimens occurred in the patients 16–24 years of age (Table 2).

Of the 93 fully genotyped specimens, six profiles appeared to have a multiple (mostly two) of VNTR sequences in one sample (Table S3), *i.e.* sequence data suggested the presence of more than one VNTR type at the same *locus* in these samples (evidenced by a succession of two nucleotides detected at the same point on the sequencing chromatogram). In one specimen, a mixed infection according to the *omp*A sequence was suspected. In this case, the *omp*A gene was cloned and sequenced, revealing that this sample contained genotypes E and D/UW-3/CX. Isolates that were cultured then genotyped belonged to genotypes D, F, G, J and K (Figure 1).

Throughout the analysis of *omp*A, various nucleotide polymorphisms in comparison to database/known sequences were noted (see Tables S4 and S5). Of the four genotype J samples, two had twelve identical nucleotide changes in the *omp*A sequence compared with J/UW-36 . Ten mismatches distinguished D/IC-CAL8 from D/UW-3 (data not shown).

**Figure 2:**
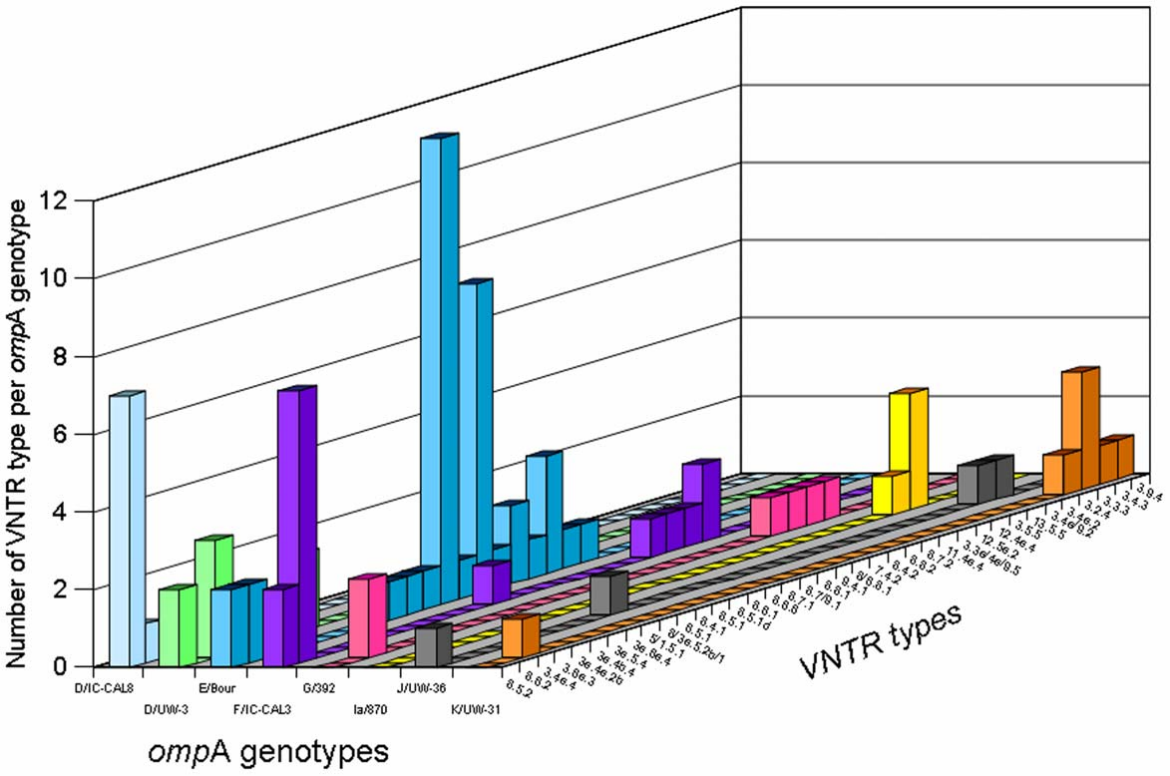
Distribution of different VNTR types according to *omp*A genotype (where *loci* appeared mixed in individual samples, both numerals are given separated by a ‘/’).

There were different MLVA types within each genotype, including the two genotype D variants (Figure 2). Overall, the combination of the *omp*A and MLVA as a genotyping panel gave, for this set of results, a Simspon's discriminatory index [15] of 0.96. Of the eight samples which could only be genotyped following culture (Table S3), five (MLVA-*omp*A types: 3.2.4-K; 3.6a.3-D/UW-3; 12.5a.2-G; 3.4a.2-J; and 8.8.2-F) were unique genotypes which had no replicates that were also detectable directly from the original DNA samples. Some MLVA signatures were common to different *omp*A types, for example 8.5.2 was found in *omp*A types D (both sub-types), E, F and J; whilst 8.6.2 was found in D/IC-CAL8, E, and F. In most cases however, MLVA signatures were unique to *omp*A types. As well as defining some new VNTR sequences for the three *loci* (Table 3), we further noted that in some cases there were differences in the flanking regions between two samples where the VNTR *locus* sequence itself was identical. For example, VNTR CT1299 variant 4 (Table 3) has a tandem repeat of ten cytosine residues (10C). In our survey, we found three versions of CT1299 variant 4 with 10C, with three different sequences between the conserved flanking regions, namely 10C (variant 4), 10C-T3C (variant 4a) and CT-10C-T3C (variant 4b). We therefore extended the signature numbering system of Pedersen et al [10] to enable differentiation by inclusion of the eight-base flanking regions. Two samples which were genotypes E and J, had a new CT1299 variant (which we have designated as variant 9; Table 3). This variant is common in LGV strains, which may indicate a closer linkage of particular genital tract strains to LGV than others.

**Table 3:** VNTR sequence analysis and description of previously unseen VNTR types.

VNTR type code (including new & modified)	CT1335 Variants^a^
1	GAAAAAG-**9T8A**-GCTTTTGT
3	GAAAAAGG-**10T8A**-GCTTTTGT
3a (modified)	GAAAAAAG-**10T8A**-GCTTTTGT
5	GAAAAAG-**11T8A**-GCTTTTGT
6	GAAAAAGG-**12T7A**-GCTTTTGT
7	GAAAAAGG-**12T8A**-GCTTTTGT
8	GAAAAAGG-**13T7A**-GCTTTTGT
9	GAAAAAGG-**13T6A**-GCTTTTGT
11 (new)	GAAAAAAG -**7T9A**-GCTTTTGT
12 (new)	GAAAAAAG -**8T9A**-GCTTTTGT
13 (new)	GAAAAAGG-**9T9A**-GCTTTTGT
	**CT1299 Variants** a
1	TTTTTATTCT-**7C**-ATCAAA
2	TTTTTATTCT-**8C**-ATCAAA
3	TTTTTATTCT-**9C**-ATCAAA
3a (modified)	TTTTTATTCT-**9C**-T3C-ATCAAA
4	TTTTTATTCT-**10C**-ATCAAA
4a (modified)	TTTTTATTCT-**10C**-T3C-ATCAAA
4b (modified)	TTTTTATTCT-CT -**10C**-T3C-ATCAAA
5	TTTTTATTCT-**11C**-ATCAAA
5a (modified)	TTTTTATTCT-**11C**-T3C-ATCAAA
6	TTTTTATTCT-**12C**-ATCAAA
6a (modified)	TTTTTATTCT-**12C**-T3C-ATCAAA
7	TTTTTATTCT-**13C**-ATCAAA
8	TTTTTATTCT-**14C**-ATCAAA
9 (new)	TTTTTATTCT-3C2T-**6C**-ATCAAA
	**CT1291 Variants Variants** a
1	AAAATGGTCTA -**6C**-TATTG **
2	AAAATGGTCT-**8C**-TATTG
2a (modified)	AAAATGGTCTA -**8C**-TATTG
2b (modified)	AAAATAGTCTA -**8C**-TATTG***
3	AAAATGGTCT-**9C**-TATTG
4	AAAATGGTCT-**10C**-TATTG
5	AAAATGGTCT-**11C**-TATTG
6 (new)	AAAATGGTCT-CT-**5C**-TATTG
7 (new)	AAAATGGTCT-**12C**-TATTG

a VNTR region shown in bold.

Flanking region variations underlined.

For each sample, *locus* CT1291 PCR mostly produced either of two different amplicon sizes (∼225bp or ∼510bp), the former amplicon being common to genotype E samples. CT1291 variant 1 (Table 3) was exclusively found in the ∼510bp amplicons. CT1291 is found within a gene coding sequence and this variation may represent a significant difference in lineages associated with variant 1 which has a large indel site between the primer-annealing sites in comparison to the variants found in the ∼225bp amplicons. Furthermore, one sample displayed evidence of deletion of *locus* CT1291.

Of the two different sizes of CT1291 PCR products (∼225bp or ∼510bp), the 225bp fragment aligned with the downstream half of the ∼510bp fragment. All ∼510bp PCR products had the same variant (variant 1; Table 3) - most found in genotype E (32 out of 34 CT1291 variant 1 samples). One sample failed to generate a VNTR sequence despite amplification of a ∼280 bp amplicon. Sequence analysis revealed this amplicon to have a sequence homologous to the upstream region only of the ∼510bp version of CT1291 PCR products.

## Discussion

This study showed that in an urban UK population, there is a greater genetic diversity among circulating *C. trachomatis* strains than can be measured by *omp*A-based genotyping alone, the (previous) gold standard for *C. trachomatis* genotyping. This revealed a diversity measured by differences in MLVA, but also showed that there is a diversity of MLVA types unique to specific *omp*A genotypes. In addition we noted that there was a diversity of *omp*A types found within certain common MLVA types, for example MLVA type 8.5.2 was distributed between *omp*A types D (both sub-types), E, F and J. This strongly suggests evidence of recombination, *i.e.* mobility of an element containing *omp*A supporting recent observations of Dean et al [4].

This report describes the first independent study of the MLVA-*omp*A genotyping scheme applied directly to routine clinical samples and used as a tool for genotyping in a survey of *C. trachomatis*-positive specimen-swabs from women. It also demonstrates that it was possible to preserve isolates (capable of undergoing more than two passages in tissue culture) based on using the swab supernatant, giving an isolation rate of 43%. An estimation of diversity according to assignment of different genotype by the MLVA-*omp*A definition indicates we have at least 26 different live variants (out of 68 culturable isolates in total) as an ongoing collection across the genotypes D, E, F, G, Ia, J and K. Further genomic analysis of these and others of the total of 68 culturable isolates may reveal a greater diversity in this collection, for example it is possible that two isolates with a MLVA-*omp*A identity of 8.7.2-F may differ significantly at other *loci*. Furthermore, the stability of VNTR *loci* should be taken into account when assigning identities. VNTR sequence may vary over only a few generations, therefore such *loci* may only be useful for once-only assessments of local epidemiology and not suitable for repeated sampling of a population. There is some evidence that these *loci* may be stable [10], but this is based upon recurrent or persistent infections in patients and not using tissue culture to grow defined generations of a single strain against which to measure stability. The incidence of genotypes that could only be cultured in this evaluation (Table S3) raises the possibility that culturing may be selective, and that certain genotypes are more suited to culture–recovery than others. The use of swab samples which employ M4RT as the transport agent for the COBAS Taqman 48 (Roche) diagnostics assay is fortuitous in that the presence of gentamicin is of benefit to chlamydial tissue culture as it helps control contamination and at the correct concentrations does not inhibit chlamydial growth [16]. It is recommended that tissue culture of isolates should be commenced within 24 hours of collection of a swab sample [17]. In our case, the time taken to allow delivery of the swabs from the GUM clinic and GP practices to the HPA Molecular Diagnostics Unit was without a reliable cold chain which adversely affected our isolation rate. Furthermore we set high criteria for isolation hence our isolate rate of 43% reflects a realistic figure with routine samples. It may be possible in future studies to obtain a greater number of isolates by freezing samples immediately after processing at −80°C so as to increase the probability of preserving viable elementary bodies.

The high numbers of samples from the 16–24 age-group (79%; Table 2) reflects the published figures for the UK South Central Strategic Health Authority (SCSHA) highlighting this to be the most at-risk age group in Southampton, which has one of the highest rates of sexually transmitted infections in the country (hpa.org.uk). In 2009 there was a 38% increase in total *C. trachomatis* cases in the SCSHA since 2000, and of the 3028 female cases in this region, 2286 fell within the 16–24 age-group.

The sampling algorithm meant that it was likely that swabs with a large load of target were mainly taken. This may have had the affect that samples arose from a proportion of the infected population which sheds large numbers of EBs, possibly symptomatic. Although the algorithm in this study was designed to ensure culturable samples, future high resolution genotyping studies should focus on DNA alone and thus bacterial load will be irrelevant, allowing more samples from asymptomatic patients to be collected.

The distribution of *omp*A genotypes was similar to that found in a survey of this marker in the UK (Cambridge) in that E was the most common genotype detected, and D and F were the next most common genotypes [18]. Interestingly, the Cambridge survey also reported no genotype H detectable from females. The abundance of genotypes D, E and F reflects many studies [19]–[21]. By contrast, Ikryannikova et al [11] showed the most abundant *omp*A genotypes to be E, G and K. This most likely reflects the very small sample size and the pre-selection of only culture-adapted strains. Culture-bias is known to underestimate certain strains in epidemiological studies, especially in difficult-to-culture pathogens [22].

Only one sample in our study was found to represent a mixed infection (*i.e.* more than one different genotype in the same sample), confirmed by cloning the amplicons into vectors and analyzing the sequences of the two separate clones obtained. The mixed infection proved to consist of genotypes equivalent to laboratory *C. trachomatis* strains D/UW-3 and E/Bour.

Comparison of *C. trachomatis* genomes has demonstrated that different chlamydia strains share >99.5% sequence identity [5], [23], therefore sequence differences are often apparent between different isolates only at the single nucleotide level. MLVA has many advantages for epidemiological studies of bacterial pathogens with highly conserved genomes [24]. The strength of MLVA is that it targets single, or small-number, nucleotide deletions or insertions in the tandem repeat *locus*. This allows for high definition differentiation between isolates in monomorphic bacterial populations but it does not indicate evolutionary relationships [25]. However, in the case of *C. trachomatis* epidemiology, MLVA helped ascertain that whilst infected partners may appear to have the same strain owing to mutual transmission according to *omp*A analysis, MLVA data showed that one partner could have the same *omp*A type but a different MLVA type, indicating links to infection outside the partnership post-mutual transmission [10]. A weakness of MLVA is that it effectively relies on “mistakes” made by the host DNA polymerase during replication, therefore although useful in a localized survey covering a selected time-period, drift in tandem repeat insertions or deletions (indels) in the same *C. trachomatis* strain may reveal disparities when comparing surveys taken at different times from the same population. A key factor in our experimental design was the choice of a high quality, high fidelity thermostable polymerase therefore we have very high confidence in the sequencing results. Our diversity index value for the MLVA-*omp*A scheme (0.96) reflects that measured by Ikryannikova et al [11] when combining the three VNTR markers and *omp*A. In our survey, although we observed a wide diversity of MLVA types particularly in genotype E, there may be drift (*e.g.* plus or minus one nucleotide of the VNTR) in the *locus* represented by the middle digit of the MLVA code. For example there were a number of results for VNTR *locus* CT1299 suggesting this might be a site which undergoes variation with replication of a single clone. This was manifested in samples which appeared to be single infections according to pure sequence data for three of the four *loci*, but with one *locus* showing evidence of several sequence variants within the tandem repeat. This was confirmed by cloning and sequencing VNTR amplicons where this phenomenon was apparent. An example of the results showed the presence of both 9C and 10C *loci* occurring in a sample with otherwise identical remaining *loci*. Examples of such single-*locus* mixed variants were found in all three VNTR loci. This could either represent genuinely different VNTR sequences, or the same VNTR sequence in the Southampton population experiencing drift of the middle *locus* (*i.e.* it is possible that the VNTR sequence in the same strain may vary by minus or plus one nucleotide with different generations). Several MLVA-*omp*A genotypes were present in large clusters indicating specific sexual transmission networks, for example there were twelve samples that were 8.5.1-E, seven 8.6.2-F, and seven 8.5.2-D/IC-CAL8 (Figure 2 and Table S3). These may represent interesting clusters of cases, that might indicate specific sexual networks in terms of lines of transmission. Future studies that would allow demographic data to be collected along with the samples may be able to answer questions regarding contact tracing (which is acknowledged as only allowing an incomplete picture of a network due to its reliance on verbal cooperation of patients [26]) using analysis of such clusters.

There are several important uses for being able to type *C. trachomatis* to a high definition once an acceptably sensitive and robust genotyping panel is available: Partner notification in tracing transmission patterns in sexual networks; connection of clinical presentation and pathogenicity to genotypes; organ and tissue tropism association with genotypes; aiding forensic investigations; discrimination of persistent infections [10] epidemiology and detection of emerging strains. To establish a reliable high resolution genotyping system, the first step is to test the system against a local population to review the fidelity of resolution [26].

Although not specified by our experimental design, all the samples were from women, most probably attributable to swabs being unpopular amongst men attending UK GUM clinics. The genotyping system deployed sequence analysis of the *omp*A gene and three VNTR *loci*. Although recent evidence reveals that *omp*A may be more mobile than previously thought [4], it remains a useful target as part of a panel of markers as in the present study because there is a limited resource of database information encompassing all possible genome-based markers for genotyping and *omp*A genes have been the most studied in terms of *C. trachomatis* epidemiology. The use of a small number of markers (*e.g.* three for VNTR) is limiting for investigating phylogenetic relationships between strains, and whilst programmes such as eBurst (http://eburst.mlst.net/) have been used the analyses appear to be of limited use [11]. However, the value of the current MLVA-*omp*A scheme seems significant based on this and other studies [10], [11] which have shown its value in determining a greater diversity in *C. trachomatis* populations than measured by *omp*A alone. Therefore further investigation and development work is required to identify more VNTR markers. High throughput analysis of chlamydia genome sequences may be the ultimate way of generating data for in-depth studies but there remain considerable challenges with this technology and sample quality [27]. A better intermediate typing scheme with greater resolution and strain definition may be possible without the need for complete genome sequencing if the target panel is increased and/or altered to include new *loci* including meaningful SNPs and markers from other schemes additional to VNTRs. Based on similar criteria the new J samples should be considered a new *omp*A genotype J subtype.

## Supporting Information

Table S1 Data for directly sequenced samples (n = 85). The samples from which *C. trachomatis* was isolated are highlight in green (column one).
(DOC)

Table S2 A: Sequencing results from 19 culturable *C. trachomatis* samples that did not return a direct MLVA- *omp*A genotype from the original clinical sample. (This data shows the eight samples that were fully genotyped after isolation in cell culture [green font – column one], and those that were rejected because they did not have good quality variable domain I and II sequence data [black font - column one].) B: The four *omp*A sequence types (column one) showing the areas of *omp*A (variable domains) where typing data was obtained. C: *omp*A types of the samples in S2A– the *omp*A sequence for sample 32 was not readable.
(DOC)

Table S3 Distribution of different VNTR types according to *omp*A genotype (total of each type).
(DOC)

Table S4 Overall distribution of *omp*A and mutations therein plus variable number tandem repeat.
(DOC)

Table S5 Nucleotide changes in *omp*A sequences.
(DOC)
