# Supplementary material for: Evaluation of a High Resolution Genotyping Method for Chlamydia trachomatis Using Routine Clinical Samples
Source: PLoS One. 2011 Feb 11;6(2):e16971. doi: 10.1371/journal.pone.0016971 (PMC3037941; doi:10.1371/journal.pone.0016971)
Supplement: Table S1 — Data for directly sequenced samples (n = 85). The samples from which C. trachomatis was isolated are highlight in green (column one). (DOC) [file pone.0016971.s001.doc]

| **No.** | **ompA** | **CT1335** | **CT1299** | **CT1291** | **VNTR type** |
| --- | --- | --- | --- | --- | --- |
| 1 | E/Bour (one nt changed) | **GAAAAAGG-13T7A-GCTTTTGT (-15nt-CCT)** | **TTTTTATTCT-12C-ATCAAA** | **AAAATGGTCTA-6C-TATTG (long frag.)** | **8.6.1** |
| 2 | E/Bour (one nt changed) | **GAAAAAGG-13T6A-GCTTTTGT (-15nt-CCT)** | **TTTTTATTCT-10C-ATCAAA** | **AAAATGGTCTA-6C-TATTG (long frag.)** | **9.4.1** |
| 3 | D/UW-3 (one nt changed) | **GAAAAAGG-13T7A-GCTTTTGT** | **TTTTTATTCT-11C-ATCAAA** | **AAAATGGTCT-8C-TATTG** | **8.5.2** |
| 5 | K/UW-31 (3nt insertions) | **GAAAAAGG-10T8A-GCTTTTGT (-15nt-CCT)** | **TTTTTATTCT-9C-ATCAAA** | **AAAATGGTCT-9C-TATTG** | **3.3.3** |
| 6 | E/Bour | **GAAAAAGG-13T7A-GCTTTTGT** | **TTTTTATTCT-12C-ATCAAA** | **AAAATGGTCT-8C-TATTG** | **8.6.2** |
| 8 | J/UW-36(11nt changed) | **GAAAAAGG-13T7A-GCTTTTGT (-15nt-CCC)** | **TTTTTATTCT-11C-ATCAAA** | **AAAATGGTCT-8C-TATTG** | **8.5.2** |
| 9 | D/IC-CAL8 | **GAAAAAGG-13T7A-GCTTTTGT** | **TTTTTATTCT-11C-ATCAAA** | **AAAATGGTCT-8C-TATTG** | **8.5.2** |
| 11 | D/IC-CAL8 | **GAAAAAGG-13T7A-GCTTTTGT (-15nt-CCC)** | **TTTTTATTCT-11C-ATCAAA** | **AAAATGGTCT-8C-TATTG** | **8.5.2** |
| 12 | F/IC-CAL3 | **GAAAAAGG-13T7A-GCTTTTGT (-15nt-CCC)** | **TTTTTATTCT-13C-ATCAAA** | **AAAATGGTCT-8C-TATTG** | **8.7.2** |
| 14 | E/Bour | **GAAAAAGG-13T7A-GCTTTTGT (-15nt-CCC)** | **TTTTTATTCT-12C-ATCAAA** | **AAAATGGTCT-8C-TATTG** | **8.6.2** |
| 17 | E/Bour | **GAAAAAGG-13T7A-GCTTTTGT (-15nt-CCT)** | **TTTTTATTCT-11C-ATCAAA** | **AAAATGGTCTA-6C-TATTG (long frag.)** | **8.5.1** |
| 19 | D/IC-CAL8 | **GAAAAAGG-13T7A-GCTTTTGT** | **TTTTTATTCT-12C-ATCAAA** | **AAAATGGTCT-8C-TATTG** | **8.6.2** |
| 20 | E/Bour | **GAAAAAGG-13T7A-GCTTTTGT** | **TTTTTATTCT-12C-ATCAAA** | **AAAATGGTCTA-6C-TATTG (long frag.)** | **8.6.1** |
| 21 | E/Bour | **GAAAAAG-11T8A/9T8A-GCTTTTGT (-15nt-CCC)** | **TTTTTATTCT-11C-ATCAAA** | **AAAATGGTCTA-6C-TATTG (long frag.)** | **5/1.5.1** |
| 22 | F/IC-CAL3 | **GAAAAAGG-13T7A-GCTTTTGT (-15nt-CCC)** | **TTTTTATTCT-12C-ATCAAA** | **AAAATGGTCT-8C-TATTG** | **8.6.2** |
| 23 | D/IC-CAL8 | **GAAAAAGG-13T7A-GCTTTTGT (-15nt-CCC)** | **TTTTTATTCT-11C-ATCAAA** | **AAAATGGTCT-8C-TATTG** | **8.5.2** |
| 24 | D/UW-3 | **GAAAAAAG-10T8A-GCTTTTGT** | **TTTTTATTCT-CT10CT3C-ATCAAA** | **AAAATGGTCT-10C-TATTG** | **3a.4b.4** |
| 25 | E/Bour | **GAAAAAGG-13T7A-GCTTTTGT** | **TTTTTATTCT-11C-ATCAAA** | No tandem repeat (recombination?) | **8.5.1d** |
| 26 | E/Bour | **GAAAAAGG-13T7A-GCTTTTGT** | **TTTTTATTCT-12C-ATCAAA** | **AAAATGGTCTA-6C-TATTG (long frag.)** | **8.6.1** |
| 30 | E/Bour | **GAAAAAGG-13T7A-GCTTTTGT** | **TTTTTATTCT-12C-ATCAAA** | **AAAATGGTCTA-6C-TATTG (long frag.)** | **8.6.1** |
| 31 | F/IC-CAL3 | **GAAAAAGG-13T7A-GCTTTTGT** | **TTTTTATTCT-11C-ATCAAA** | **AAAATGGTCTA-6C-TATTG (long frag.)** | **8.5.1** |
| 36 | G/392 | **GAAAAAGG-10T8A-GCTTTTGT** | mix of 3C2T6C/10C1T3C/9C1T3C? | **AAAATGGTCT-11C-TATTG** | **3.3a/4a/9.5** |
| 41 | F/IC-CAL3 | **GAAAAAGG-13T7A-GCTTTTGT** | **TTTTTATTCT-13C-ATCAAA** | **AAAATGGTCT-8C-TATTG** | **8.7.2** |
| 42 | D/UW-3 | **GAAAAAGG-10T8A-GCTTTTGT** | **TTTTTATTCT-10C1T3C-ATCAAA** | **AAAATGGTCT-10C-TATTG** | **3.4a.4** |
| 44 | D/UW-3 | **GAAAAAGG-13T7A-GCTTTTGT** | **TTTTTATTCT-11C-ATCAAA** | **AAAATGGTCT-8C-TATTG** | **8.5.2** |
| 45 | F/IC-CAL3 | **GAAAAAGG-13T7A-GCTTTTGT** | **TTTTTATTCT-11C-ATCAAA** | **AAAATGGTCT-8C-TATTG** | **8.5.2** |
| 48 | F/IC-CAL3 | **GAAAAAGG-13T7A-GCTTTTGT** | **TTTTTATTCT-12C-ATCAAA** | **AAAATGGTCT-8C-TATTG** | **8.6.2** |
| 49 | D/IC-CAL8 | **GAAAAAGG-13T7A-GCTTTTGT** | **TTTTTATTCT-11C-ATCAAA** | **AAAATGGTCT-8C-TATTG** | **8.5.2** |
| 51 | E/Bour | **GAAAAAGG-13T7A-GCTTTTGT** | **TTTTTATTCT-12C-ATCAAA** | **AAAATGGTCTA-6C-TATTG (long frag.)** | **8.6.1** |
| 52 | E/Bour | **GAAAAAGG-13T7A-GCTTTTGT** | **TTTTTATTCT-12C-ATCAAA** | **AAAATGGTCT-CTCCCCC-TATTG** | **8.6.6** |
| 53 | E/Bour | **GAAAAAGG-13T7A-GCTTTTGT** | **TTTTTATTCT-11C-ATCAAA** | **AAAATGGTCTA-6C-TATTG (long frag.)** | **8.5.1** |
| 54 | D/UW-3 | **GAAAAAGG-10T8A-GCTTTTGT (-15nt-CCC)** | **TTTTTATTCT-10C1T3C-ATCAAA** | **AAAATGGTCT-10C-TATTG** | **3.4a.4** |
| 56 | J/UW-36 | **GAAAAAGG-10T8A-GCTTTTGT (-15nt-CCC)** | mix of 3C2T6C and 10C1T3C? | **AAAATGGTCT-8C-TATTG** | **3.4a/9.2** |
| 60 | E/Bour | **GAAAAAGG-13T7A-GCTTTTGT** | **TTTTTATTCT-13C-ATCAAA** | **AAAATGGTCTA-6C-TATTG (long frag.)** | **8.7.1** |
| 65 | D/IC-CAL8 | **GAAAAAGG-13T7A-GCTTTTGT (-15nt-CCC)** | **TTTTTATTCT-11C-ATCAAA** | **AAAATGGTCT-8C-TATTG** | **8.5.2** |
| 67 | E/Bour | **GAAAAAGG-13T7A-GCTTTTGT (-15nt-CCT)** | **TTTTTATTCT-14C-ATCAAA** | **AAAATGGTCTA-6C-TATTG (long frag.)** | **8.8.1** |
| 69 | D/UW-3 | **GAAAAAAG-10T8A-GCTTTTGT (-15nt-CCC)** | **TTTTTATTCT-11C-ATCAAA** | **AAAATGGTCT-10C-TATTG** | **3a.5.4** |
| 70 | E/Bour | **GAAAAAGG-13T7A-GCTTTTGT** | **TTTTTATTCT-12C-ATCAAA** | **AAAATGGTCTA-6C-TATTG (long frag.)** | **8.6.1** |
| 75 | F/IC-CAL3 | **GAAAAAGG-13T7A-GCTTTTGT** | **TTTTTATTCT-10C-ATCAAA** | **AAAATGGTCT-8C-TATTG** | **8.4.2** |
| 79 | G/392 | **GAAAAAAG-7T9A-GCTTTTGT (-15nt-CCC)** | **TTTTTATTCT-10C1T3C-ATCAAA** | **AAAATGGTCT-10C-TATTG** | **11.4a.4** |
| 80 | K/UW-31 | **GAAAAAGG-10T8A-GCTTTTGT (-15nt-CCC)** | **TTTTTATTCT-10C1T3C-ATCAAA** | **AAAATGGTCT-10C-TATTG** | **3.4a.4** |
| 81 | J/UW-36 (>10 mis-match) | **GAAAAAGG-12T7A-GCTTTTGT (-15nt-CCT)** | **TTTTTATTCT-11C-ATCAAA** | **AAAATGGTCTA-6C-TATTG (long frag.)** | **6.5.1** |
| 83 | E/Bour | **GAAAAAGG-13T7A-GCTTTTGT** | **TTTTTATTCT-11C-ATCAAA** | **AAAATGGTCT-8C-TATTG** | **8.5.2** |
| 84 | E/Bour | **GAAAAAGG-13T7A-GCTTTTGT (-15nt-CCT)** | **TTTTTATTCT-11C-ATCAAA** | **AAAATGGTCTA-6C-TATTG (long frag.)** | **8.5.1** |
| 91 | E/Bour | **GAAAAAGG-13T7A-GCTTTTGT (-15nt-CCT)** | **TTTTTATTCT-11C-ATCAAA** | **AAAATGGTCTA-6C-TATTG (long frag.)** | **8.5.1** |
| 93 | Ia/870 | **GAAAAAGG-9T9A-GCTTTTGT (-15nt-CCC)** | **TTTTTATTCT-11C-ATCAAA** | **AAAATGGTCT-11C-TATTG** | **13.5.5** |
| 94 | E/Bour | **GAAAAAGG-13T7A-GCTTTTGT (-15nt-CCT)** | **TTTTTATTCT-13C-ATCAAA** | **AAAATGGTCTA-6C-TATTG (long frag.)** | **8.7.1** |
| 96 | E/Bour | **GAAAAAGG-12T7A-GCTTTTGT** | **TTTTTATTCT-11C-ATCAAA** | **AAAATGGTCTA-6C-TATTG (long frag.)** | **6.5.1** |
| 97 | F/IC-CAL3 | **GAAAAAGG-13T7A-GCTTTTGT** | **TTTTTATTCT-11C-ATCAAA** | **AAAATGGTCT-8C-TATTG** | **8.5.2** |
| 101 | E/Bour and D/UW-3 mix | **GAAAAAGG-13T7A/12T7A-GCTTTTGT** | **TTTTTATTCT-14C-ATCAAA** | **AAAATGGTCTA-6C-TATTG (long frag.)** | **8/6.8.1** |
| 106 | F/IC-CAL3 | **GAAAAAGG-13T7A-GCTTTTGT (-15nt-CCC)** | **TTTTTATTCT-12C-ATCAAA** | **AAAATGGTCT-8C-TATTG** | **8.6.2** |
| 107 | E/Bour | **GAAAAAGG-13T7A-GCTTTTGT (-15nt-CCT)** | **TTTTTATTCT-11C-ATCAAA** | **AAAATGGTCTA-6C-TATTG (long frag.)** | **8.5.1** |
| 108 | E/Bour | **GAAAAAGG-13T7A-GCTTTTGT** | **TTTTTATTCT-11C-ATCAAA** | **AAAATGGTCT-8C-TATTG** | **8.5.2** |
| 109 | K/UW-31 | **GAAAAAGG-10T8A-GCTTTTGT (-15nt-CCC)** | **TTTTTATTCT-9C-ATCAAA** | **AAAATGGTCT-9C-TATTG** | **3.3.3** |
| 112 | E/Bour | **GAAAAAGG-13T7A-GCTTTTGT** | mix of 3C2T6C and 13C? | **AAAATGGTCTA-6C-TATTG (long frag.)** | **8.7/9.1** |
| 114 | E/Bour | **GAAAAAGG-13T7A-GCTTTTGT** | **TTTTTATTCT-11C-ATCAAA** | **AAAATGGTCTA-6C-TATTG (long frag.)** | **8.5.1** |
| 116 | E/Bour | **GAAAAAGG-13T7A-GCTTTTGT** | **TTTTTATTCT-11C-ATCAAA** | **AAAATGGTCTA-6C-TATTG (long frag.)** | **8.5.1** |
| 117 | D/UW-3 | **GAAAAAAG-10T8A-GCTTTTGT** | **TTTTTATTCT-10CT3C-ATCAAA** | **AAAATAGTCTA-8C-TATTG** | **3a.4a.2b** |
| 118 | F/IC-CAL3 | **GAAAAAGG-13T7A-GCTTTTGT** | **TTTTTATTCT-12C-ATCAAA** | **AAAATGGTCT-8C-TATTG** | **8.6.2** |
| 119 | E/Bour | **GAAAAAGG-13T7A-GCTTTTGT** | **TTTTTATTCT-14C-ATCAAA** | **AAAATGGTCTA-6C-TATTG (long frag.)** | **8.8.1** |
| 120 | E/Bour | **GAAAAAGG-13T7A-GCTTTTGT (-15nt-CCT)** | **TTTTTATTCT-11C-ATCAAA** | **AAAATGGTCTA-6C-TATTG (long frag.)** | **8.5.1** |
| 124 | E/Bour | **GAAAAAGG-13T7A-GCTTTTGT** | **TTTTTATTCT-14C-ATCAAA** | **AAAATGGTCTA-6C-TATTG (long frag.)** | **8.8.1** |
| 126 | D/UW-3 | **GAAAAAGG-10T8A-GCTTTTGT** | **TTTTTATTCT-10CT3C-ATCAAA** | **AAAATGGTCT-10C-TATTG** | **3.4a.4** |
| 127 | E/Bour | **GAAAAAGG-13T7A-GCTTTTGT** | **TTTTTATTCT-11C-ATCAAA** | **AAAATGGTCTA-6C-TATTG (long frag.)** | **8.5.1** |
| 128 | D/IC-CAL8 | **GAAAAAGG-13T7A-GCTTTTGT** | **TTTTTATTCT-11C-ATCAAA** | **AAAATGGTCT-8C-TATTG** | **8.5.2** |
| 129 | E/Bour | **GAAAAAGG-13T7A-GCTTTTGT** | **TTTTTATTCT-11C-ATCAAA** | **AAAATGGTCTA-6C-TATTG (long frag.)** | **8.5.1** |
| 130 | D/UW-3 | **GAAAAAAG-10T8A-GCTTTTGT (-15nt-CCC)** | **TTTTTATTCT-12CT3C-ATCAAA** | **AAAATGGTCT-10C-TATTG** | **3a.6a.4** |
| 131 | D/UW-3 | **GAAAAAAG-10T8A-GCTTTTGT** | **TTTTTATTCT-12CT3C-ATCAAA** | **AAAATGGTCT-10C-TATTG** | **3a.6a.4** |
| 132 | Ia/870 | **GAAAAAGG-10T8A-GCTTTTGT** | **TTTTTATTCT-11C-ATCAAA** | **AAAATGGTCT-11C-TATTG** | **3.5.5** |
| 135 | G/392 (one nt changed) | **GAAAAAAG-8T9A-GCTTTTGT** | **TTTTTATTCT-10CT3C-ATCAAA** | **AAAATGGTCT-10C-TATTG** | **12.4a.4** |
| 136 | K/UW-31 | **GAAAAAGG-10T8A-GCTTTTGT** | **TTTTTATTCT-3C2T6C-ATCAAA** | **AAAATGGTCT-10C-TATTG** | **3.9.4** |
| 137 | F/IC-CAL3 | **GAAAAAGG-13T7A-GCTTTTGT** (mix with 10T8A?) | **TTTTTATTCT-12C-ATCAAA** | **AAAATGGTCT-8C-TATTG** | **8.6.2** |
| 138 | Ia/870 | **GAAAAAGG-9T9A-GCTTTTGT** | **TTTTTATTCT-11C-ATCAAA** | **AAAATGGTCT-11C-TATTG** | **13.5.5** |
| 139 | G/392 | **GAAAAAGG-10T8A-GCTTTTGT** | **TTTTTATTCT-10CT3C-ATCAAA** | **AAAATGGTCT-10C-TATTG** | **3.4a.4** |
| 140 | F/IC-CAL3 | **GAAAAAGG-12T8A-GCTTTTGT** | **TTTTTATTCT-10C-ATCAAA** | **AAAATGGTCT-8C-TATTG** | **7.4.2** |
| 144 | G/392 | **GAAAAAGG-10T8A-GCTTTTGT (-15nt-CCC)** | **TTTTTATTCT-10CT3C-ATCAAA** | **AAAATGGTCT-10C-TATTG** | **3.4a.4** |
| 145 | E/Bour | **GAAAAAGG-13T7A-GCTTTTGT** | **TTTTTATTCT-11C-ATCAAA** | **AAAATGGTCTA-6C-TATTG (long frag.)** | **8.5.1** |
| 146 | E/Bour | **GAAAAAGG-13T7A-GCTTTTGT** | **TTTTTATTCT-10C-ATCAAA** | **AAAATGGTCTA-6C-TATTG (long frag.)** | **8.4.1** |
| 148 | F/IC-CAL3 | **GAAAAAGG-13T7A-GCTTTTGT (-15nt-CCC)** | **TTTTTATTCT-12C-ATCAAA** | **AAAATGGTCT-8C-TATTG** | **8.6.2** |
| 151 | E/Bour | **GAAAAAGG-13T7A-GCTTTTGT** | **TTTTTATTCT-12C-ATCAAA** | **AAAATGGTCTA-6C-TATTG (long frag.)** | **8.6.1** |
| 152 | E/Bour | **GAAAAAGG-13T7A-GCTTTTGT** | **TTTTTATTCT-12C-ATCAAA** | **AAAATGGTCTA-6C-TATTG (long frag.)** | **8.6.1** |
| 154 | K/UW-31 | **GAAAAAGG-10T8A-GCTTTTGT (-15nt-CCC)** | **TTTTTATTCT-10C-ATCAAA** | **AAAATGGTCT-9C-TATTG** | **3.4.3** |
| 156 | Ia/870 | **GAAAAAGG-9T9A-GCTTTTGT** | **TTTTTATTCT-11C-ATCAAA** | **AAAATGGTCT-11C-TATTG** | **13.5.5** |
| 157 | E/Bour | **GAAAAAGG-13T7A-GCTTTTGT mixed with GAAAAAAG-10T8A-GCTTTTGT** | **TTTTTATTCT-11C-ATCAAA** | **AAAATAGTCTA-8C-TATTG** | **8/3a.5.2b/1** |
| 159 | E/Bour | **GAAAAAGG-13T7A-GCTTTTGT (-15nt-CCT)** | **TTTTTATTCT-11C-ATCAAA** | **AAAATGGTCTA-6C-TATTG (long frag.)** | **8.5.1** |
