# Supplementary material for: Evaluation of a High Resolution Genotyping Method for Chlamydia trachomatis Using Routine Clinical Samples
Source: PLoS One. 2011 Feb 11;6(2):e16971. doi: 10.1371/journal.pone.0016971 (PMC3037941; doi:10.1371/journal.pone.0016971)
Supplement: Table S2 — A: Sequencing results from 19 culturable C. trachomatis samples that did not return a direct MLVA- ompA genotype from the original clinical sample. (This data shows the eight samples that were fully genotyped after isolation in cell culture [green font – column one], and those that were rejected because they did not have good quality variable domain I and II sequence data [black font - column one].) B: The four ompA sequence types (column one) showing the areas of ompA (variable domains) where typing data was obtained. C: ompA types of the samples in S2A– the ompA sequence for sample 32 was not readable. (DOC) [file pone.0016971.s002.doc]

Table S2A

| **No.** | **CT1291 seq (AAAATGGTCT-CT1291-TATTG)** | **CT1299 seq (TTTTTATTCT-CT1299-ATCAAA)** | **CT1335 seq (GAAAAAGG-CT1335-GCTTTTGT)** | ***omp*A seq**  **(cover VDI &II or VDIII)** |
| --- | --- | --- | --- | --- |
|  |  |  |  | **(see below for details)** |
| **13** | **AAAATGGTCT-10C-TATTG** | **TTTTTATTCT-9C-ATCAAA** | **GAAAAAGG-10T8A-GCTTTTGT (-15nt-CCC)** | K/UW-31 |
| **15** | **AAAATGGTCT-8C-TATTG** | **TTTTTATTCT-11C-ATCAAA** | **GAAAAAGG-13T7A-GCTTTTGT (-15nt-CCC)** | D/IC-CAL8 |
| **18** | **AAAATGGTCT-8C-TATTG** | **TTTTTATTCT-12C-ATCAAA** | **GAAAAAGG-13T7A-GCTTTTGT (-15nt-CCC)** | F/IC-CAL3 |
| **32** | **AAAATGGTCT-9C-TATTG** | **TTTTTATTCT-8C-ATCAAA** | **GAAAAAGG-10T8A-GCTTTTGT (-15nt-CCC)** | **- (not readable)** |
| **34** | **AAAATGGTCT-11C-TATTG** | **TTTTTATTCT-11C-ATCAAA** | **GAAAAAGG-9T9A-GCTTTTGT (-15nt-CCC)** | Ia/870 |
| **37** | **AAAATGGTCT-10C-TATTG** | **TTTTTATTCT-8C-ATCAAA** | **GAAAAAGG-10T8A-GCTTTTGT (-15nt-CCC)** | K/UW-31 |
| **47** | **AAAATGGTCT-9C-TATTG** | **TTTTTATTCT-12CT3C-ATCAAA** | **GAAAAAGG-10T8A-GCTTTTGT (-15nt-CCC)** | D/UW-3 |
| **57** | **AAAATGGTCT-8C-TATTG** | **TTTTTATTCT-11CT3C-ATCAAA** | **GAAAAAAG-8T9A-GCTTTTGT (-15nt-CCC)** | G/392 (1nt changed) |
| **63** | **AAAATGGTCT-9C-TATTG** | **TTTTTATTCT-9C-ATCAAA** | **GAAAAAGG-10T8A-GCTTTTGT (-15nt-CCC)** | K/UW-31 |
| **72** | **AAAATGGTCT-10C-TATTG** | **TTTTTATTCT-11CT3C-ATCAAA** | **GAAAAAGG-10T8A-GCTTTTGT (-15nt-CCC)** | J/UW-36 |
| **73** | **No PCR** | **TTTTTATTCT-11C-ATCAAA** | **GAAAAAGG-13T7A-GCTTTTGT (-15nt-CCT)** | E/Bour |
| **87** | **AAAATGGTCT-8C-TATTG** | **TTTTTATTCT-10CT3C-ATCAAA** | **GAAAAAGG-10T8A-GCTTTTGT (-15nt-CCC)** | J/UW-36 (1nt changed) |
| **88** | **AAAATGGTCT-8C-TATTG** | **TTTTTATTCT-14C-ATCAAA** | **GAAAAAGG-13T7A-GCTTTTGT (-15nt-CCC)** | F/IC-CAL3 |
| **98** | **AAAATGGTCT-10C-TATTG** | **TTTTTATTCT-7C-ATCAAA** | **GAAAAAGG-10T8A-GCTTTTGT (-15nt-CCC)** | J/UW-36 |
| **103** | **AAAATGGTCT-12C-TATTG** | **TTTTTATTCT-11CT3C-ATCAAA** | **GAAAAAGG-10T8A-GCTTTTGT (-15nt-CCC)** | Ia/870 |
| **121** | **- (not readable)** | **TTTTTATTCT-12C-ATCAAA** | **GAAAAAGG-13T7A-GCTTTTGT (-15nt-CCT)** | E/Bour |
| **122** | **AAAATGGTCTA-6C-TATTG** | **TTTTTATTCT-11C-ATCAAA** | **GAAAAAGG-13T7A-GCTTTTGT (-15nt-CCT)** | E/Bour |
| **150** | **AAAATGGTCT-11C-TATTG** | **TTTTTATTCT-10CT3C-ATCAAA** | **GAAAAAGG-10T8A-GCTTTTGT (-15nt-CCC)** | D/UW-3 |
| **155** | **AAAATAGTCTA-8C-TATTG** | **TTTTTATTCT-11C-ATCAAA** | **GAAAAAGG-13T7A-GCTTTTGT (-15nt-CCT)** | E/Bour |

Table S2B

| **Type** | **Seq length**  **460 bases** | **Seq length**  **700 bases** | **Seq quality**  **1-460** | **Seq quality**  **470-700** | **Aligned seq (cover the region)** | **samples** |
| --- | --- | --- | --- | --- | --- | --- |
| **1** | **+++** | **-** | **good** |  | ***omp*A rc (VDI&II)** | **15, 18, 88** |
| **2** | **-** | **+++** | **good** | **good** | ***omp*A (VDI, II& III)** | **122, 150, 155** |
| **3** | **+++** | **+** | **Slightly mix** | **good** | ***omp*A rc (VDI&II) and *omp*A (VDIII)** | **37, 47, 57, 63, 73, 87** |
| **4** | **+++** | **+++** | **mix** | **good** | ***omp*A (VDIII)** | **13, 34, 72, 98, 103, 121** |

**Table S2C**

| **Sample No.** | **13** | **15** | **18** | **34** | **37** | **47** | **57** | **63** | **72** |
| --- | --- | --- | --- | --- | --- | --- | --- | --- | --- |
| Serovar type | K/UW-31(?) | D/IC-CAL8 | F/IC-CAL3 | Ia/870(?) | K/UW-31 | D/UW-3 | G/392 | K/UW-31 | J/UW-36(?) |
| VD region confirmed | VDIII | VDI&II | VDI&II | VDIII | VDI,II&III | VDI,II&III | VDI,II&III | VDI,II&III | VDIII |
| **Sample No.** | **73** | **87** | **88** | **98** | **103** | **121** | **122** | **150** | **155** |
| Serovar type | E/Bour | J/UW-36 | F/IC-CAL3 | J/UW-36(?) | Ia/870(?) | E/Bour(?) | E/Bour | D/UW-3 | E/Bour |
| VD region confirmed | VDI,II&III | VDI,II&III | VDI&II | VDIII | VDIII | VDIII | VDI,II&III | VDI,II&III | VDI,II&III |
