# Supplementary material for: Evaluation of a High Resolution Genotyping Method for Chlamydia trachomatis Using Routine Clinical Samples
Source: PLoS One. 2011 Feb 11;6(2):e16971. doi: 10.1371/journal.pone.0016971 (PMC3037941; doi:10.1371/journal.pone.0016971)
Supplement: Table S3 — Distribution of different VNTR types according to ompA genotype (total of each type). (DOC) [file pone.0016971.s003.doc]

| ***omp*A type:** | **D/IC-CAL8** | **D/UW-3** | **E/Bour** | **F/IC-CAL3** | **G/392** | **Ia/870** | **J/UW-36** | **K/UW-31** |
| --- | --- | --- | --- | --- | --- | --- | --- | --- |
|  |  |  |  |  |  |  |  |  |
| **VNTR types** | 8.5.2 (7*) | 8.5.2 (2) | 8.5.2 (2) | 8.5.2 (2) |  |  | 8.5.2 (1) |  |
|  | 8.6.2 (1) |  | 8.6.2 (2) | 8.6.2 (7*) |  |  |  |  |
|  |  | 3.4a.4 (3) |  |  | 3.4a.4 (2) |  |  | 3.4a.4 (1) |
|  |  | 3.6a.3 (1*) |  |  |  |  |  |  |
|  |  | 3a.4a.2b (1) |  |  |  |  |  |  |
|  |  | 3a.4b.4 (1) |  |  |  |  |  |  |
|  |  | 3a.5.4 (1) |  |  |  |  |  |  |
|  |  | 3a.6a.4 (2) |  |  |  |  |  |  |
|  |  |  | 5/1.5.1 (1) |  |  |  |  |  |
|  |  |  | 8/3a.5.2b/1 (1) |  |  |  |  |  |
|  |  |  | 6.5.1 (1) |  |  |  | 6.5.1 (1) |  |
|  |  |  | 8.4.1 (1) |  |  |  |  |  |
|  |  |  | 8.5.1 (12) | 8.5.1 (1) |  |  |  |  |
|  |  |  | 8.5.1d (1) |  |  |  |  |  |
|  |  |  | 8.6.1 (8) |  |  |  |  |  |
|  |  |  | 8.6.6 (1) |  |  |  |  |  |
|  |  |  | 8.7.1 (2) |  |  |  |  |  |
|  |  |  | 8.7/9.1 (1) |  |  |  |  |  |
|  |  |  | 8.8.1 (3) |  |  |  |  |  |
|  |  |  | 9.4.1 (1) |  |  |  |  |  |
|  |  |  | 8/6.8.1 (1) |  |  |  |  |  |
|  |  |  |  | 7.4.2 (1) |  |  |  |  |
|  |  |  |  | 8.4.2 (1) |  |  |  |  |
|  |  |  |  | 8.8.2 (1*) |  |  |  |  |
|  |  |  |  | 8.7.2 (2) |  |  |  |  |
|  |  |  |  |  | 11.4a.4 (1) |  |  |  |
|  |  |  |  |  | 3.3a/4a/9.5 (1) |  |  |  |
|  |  |  |  |  | 12.5a.2 (1*) |  |  |  |
|  |  |  |  |  | 12.4a.4 (1) |  |  |  |
|  |  |  |  |  |  | 3.5.5 (1) |  |  |
|  |  |  |  |  |  | 13.5.5 (3) |  |  |
|  |  |  |  |  |  |  | 3.4a/9.2 (1) |  |
|  |  |  |  |  |  |  | 3.4a.2 (1*) |  |
|  |  |  |  |  |  |  |  | 3.2.4 (1*) |
|  |  |  |  |  |  |  |  | 3.3.3 (3*) |
|  |  |  |  |  |  |  |  | 3.4.3 (1) |
|  |  |  |  |  |  |  |  | 3.9.4 (1) |

*= one of the isolates genotyped post-culture only

n/n = more than one apparent VNTR sequence at a specific site
