# Supplementary material for: Evaluation of a High Resolution Genotyping Method for Chlamydia trachomatis Using Routine Clinical Samples
Source: PLoS One. 2011 Feb 11;6(2):e16971. doi: 10.1371/journal.pone.0016971 (PMC3037941; doi:10.1371/journal.pone.0016971)
Supplement: Table S4 — Overall distribution of ompA and mutations therein plus variable number tandem repeat. (DOC) [file pone.0016971.s004.doc]

| ***omp*A type (mutations)** | **VNTR type** | **No. of samples** | **Culture positive** |
| --- | --- | --- | --- |
| **D/IC-CAL8** | **8.5.2** | **6** | **3** |
| **D/IC-CAL8** | **8.6.2** | **1** | **0** |
| **D/UW-3 (841CtoT)** | **8.5.2** | **1** | **0** |
| **D/UW-3 (977CtoT)** | **8.5.2** | **1** | **1** |
| **D/UW-3** | **3a.4a.2b** | **1** | **0** |
| **D/UW-3** | **3.4a.4** | **3** | **3** |
| **D/UW-3** | **3a.4b.4** | **1** | **1** |
| **D/UW-3** | **3a.5.4** | **1** | **1** |
| **D/UW-3** | **3a.6a.4** | **2** | **1** |
| **E/Bour** | **8.6.6** | **1** | **0** |
| **E/Bour** | **8.5.2** | **2** | **2** |
| **E/Bour** | **8.6.2** | **2** | **1** |
| **E/Bour** | **6.5.1** | **1** | **0** |
| **E/Bour** | **8/3a.5.2b/1** | **1** | **0** |
| **E/Bour** | **8.5.1d** | **1** | **0** |
| **E/Bour (144CtoT)** | **9.4.1** | **1** | **0** |
| **E/Bour** | **8.4.1** | **1** | **1** |
| **E/Bour** | **5/1.5.1** | **1** | **0** |
| **E/Bour** | **6.5.1** | **1** | **1** |
| **E/Bour** | **8.5.1** | **12** | **8** |
| **E/Bour** | **8.6.1** | **6** | **2** |
| **E/Bour (934GtoA)** | **8.6.1** | **2** | **0** |
| **E/Bour** | **8.7.1** | **2** | **2** |
| **E/Bour** | **8.8.1** | **3** | **2** |
| **E/Bour (mix with D)** | **8/9.8.1** | **1** | **1** |
| **E/Bour** | **8.9/7.1** | **1** | **0** |
| **F/IC-CAL3** | **7.4.2** | **1** | **0** |
| **F/IC-CAL3** | **8.4.2** | **1** | **1** |
| **F/IC-CAL3** | **8.5.2** | **2** | **1** |
| **F/IC-CAL3** | **8.6.2** | **6** | **5** |
| **F/IC-CAL3** | **8.7.2** | **2** | **2** |
| **F/IC-CAL3** | **8.5.1** | **1** | **0** |
| **G/392 (2nts changed)** | **10.4a.4** | **1** | **1** |
| **G/392 (1003GtoT)** | **11.4a.4** | **1** | **1** |
| **G/392 (1003GtoT)** | **3.4a.4** | **2** | **2** |
| **G/392 (4nts changed)** | **3.3a/4a/9.5** | **1** | **1** |
| **Ia/870** | **12.5.5** | **3** | **2** |
| **Ia/870** | **3.5.5** | **1** | **1** |
| **J/UW-36 (12 nts changed)** | **8.5.2** | **1** | **0** |
| **J/UW-36 (12 nts changed)** | **6.5.1** | **1** | **0** |
| **J/UW-36** | **3.4a/9.2** | **1** | **1** |
| **K/UW-31 (3nts insertion?)** | **3.3.3** | **1** | **0** |
| **K/UW-31** | **3.3.3** | **1** | **0** |
| **K/UW-31** | **3.4.3** | **1** | **0** |
| **K/UW-31** | **3.4a.4** | **1** | **1** |
| **K/UW-31** | **3.9.4** | **1** | **0** |
