# Supplementary material for: Evaluation of a High Resolution Genotyping Method for Chlamydia trachomatis Using Routine Clinical Samples
Source: PLoS One. 2011 Feb 11;6(2):e16971. doi: 10.1371/journal.pone.0016971 (PMC3037941; doi:10.1371/journal.pone.0016971)
Supplement: Table S5 — Nucleotide changes in ompA sequences. (DOC) [file pone.0016971.s005.doc]

| **Comparative genotype**  **(accession, no. & length)** | **Unlinked anonymised specimen no.** | **Number of nucleotides changed** | **Position in *omp*A** (according to D/UW-3/CX, accession number NC 000117) | **Amino acid code change (amino acid)** |
| --- | --- | --- | --- | --- |
| D/UW-3 (DQ064284, 1182 bp) | 3 | 1 | 841 C🡪T | CTG(Leu)🡪TTG(Leu) |
|  | 44 | 1 | 977 C🡪T | GCT(Ala)🡪GTT(Val) |
| E/Bour (DQ064286, 1182 bp) | 1  51 | 1 | 934 G🡪A | GCT(Ala)🡪ACT(Thr) |
|  | 2 | 1 | 144 C🡪T | TCC(Cys)🡪TCT(Ser) |
| G/392 (DQ064288, 1188 bp) | 36  (Same as in Pedersen’s paper, Partner 20 & 21) | 4 | 228 T🡪A,  487 G🡪A,  700 G🡪C,  1003 G🡪A | ACT(Thr)🡪ACA(Thr)  GGT(Gly)🡪AGT(Ser)  GAG(Glu)🡪CAG(Gln)  GCG(Ala)🡪ACG(Thr) |
|  | 79 | 2 | 487 G🡪A,  1003 G🡪T | GGT(Gly)🡪AGT(Ser)  GCG(Ala)🡪TCG(Ser) |
|  | 135  139  144 | 1 | 1003 G🡪T  (In Pedersen’s paper, original 1003nt=T) | GCG(Ala)🡪TCG(Ser) |
| J/UW-36 (DQ064292, 1194 bp) | 8  81 | 12 | 268 A🡪G, 269 C🡪T,  310 G🡪A,  499 G🡪T,  522 T🡪G,  526 T🡪A,  555 G🡪A,  681 G🡪A,  684 A🡪G,  813 C🡪T,  997 G🡪A,  1020 C🡪A | ACC(Thr)🡪GTC(Val)  GTT(Val)🡪ATT(Ile)  GCT(Ala)🡪TCT(Ser)  AAT(Asn)🡪AAG(Lys)  TTT(Phe)🡪ATT(Ile)  GTG(Val)🡪GTA(Val)  GAG(Glu)🡪GAA(Glu)  TTA(Leu)🡪TTG(Leu)  TAC(Tyr)🡪TAT(Tyr)  GTC(Val)🡪ATC(Ile)  GAC(Asp)🡪GAA(Glu) |
| K/UW-31 (DQ064293, 1194 bp) | 5 | 3 insertions (?)  (not convincing) | 405 insert T(after 4T)  398 insert G(after 3G)  390 insert A(after 3A) | ompA_5 readable sequence is shorter |
| D/UW-3 & E/Bour | 101 | 2 change in D? | 269 C🡪G  841 C🡪T | ACT(Thr)🡪AGT(Ser)  CTG(Leu)🡪TTG(Leu) |
